# Delayed Myelin Oligodendrocyte Glycoprotein Antibody-Associated Demyelination Five Years After an Acute Disseminated Encephalomyelitis-Like Episode in an Adolescent: A Case Report

**DOI:** 10.7759/cureus.111873

**Published:** 2026-07-01

**Authors:** Hind Zahiri, Aziza Elouali, Abdeladim Babakhouya, Maria Rkain

**Affiliations:** 1 Pediatric Neurology, Mohammed VI University Hospital, Faculty of Medicine and Pharmacy, Mohammed First University, Oujda, MAR; 2 Pediatrics, Mohammed VI University Hospital, Faculty of Medicine and Pharmacy, Mohammed First University, Oujda, MAR; 3 Pediatric Gastroenterology, Hospital Center University Mohammed VI, Oujda, MAR

**Keywords:** acute disseminated encephalomyelitis (adem), cell-based assay, delayed relapse, longitudinally extensive transverse myelitis, mog-igg, mycophenolate mofetil (mmf), myelin oligodendrocyte glycoprotein antibody-associated disease (mogad), pediatric demyelinating disease, pediatric neuroimmunology

## Abstract

Myelin oligodendrocyte glycoprotein antibody-associated disease (MOGAD) is an immune-mediated inflammatory demyelinating disorder of the central nervous system with a broad pediatric clinical spectrum. Acute disseminated encephalomyelitis (ADEM)-like presentations are more frequent in younger children, whereas older children and adolescents may present with optic neuritis, transverse myelitis, or multifocal central nervous system involvement. We report the case of a 14-year-old male adolescent who developed a severe MOG-IgG-positive demyelinating event five years after an initial ADEM-like episode. At nine years of age, he presented with back pain, gait disturbance, urinary incontinence, mild drowsiness, fever, cerebrospinal fluid pleocytosis, subcortical and medullary brain lesions, and longitudinally extensive myelitis. MOG-IgG testing was not available at that time, and the episode was diagnosed as ADEM based on the overall clinico-radiological presentation, with complete recovery after corticosteroid therapy. Five years later, he presented with fever, acute quadriparesis predominating in the lower limbs, pyramidal signs, and sphincter dysfunction. Brain MRI showed multiple ill-defined subcortical T2-weighted/fluid-attenuated inversion recovery (T2/FLAIR) hyperintense lesions, and spinal MRI demonstrated longitudinally extensive cervical myelitis from the cervicomedullary junction to C6. Cerebrospinal fluid analysis showed marked pleocytosis, elevated protein, absence of oligoclonal bands, and negative infectious studies. Serum MOG-IgG was positive at a titer of 1:100 using a fixed cell-based assay, while AQP4-IgG was negative. He received intravenous methylprednisolone followed by intravenous immunoglobulin because of insufficient initial improvement, with complete clinical recovery at one month and marked radiological regression at six months. This case emphasizes that a confirmed MOG-IgG-positive demyelinating event may occur after a prolonged relapse-free interval following an earlier ADEM-like demyelinating episode, while also underscoring that the first episode should be interpreted cautiously because MOG-IgG testing was unavailable. Long-term follow-up remains essential because short-term clinical and radiological improvement does not establish durable disease control.

## Introduction

Myelin oligodendrocyte glycoprotein antibody-associated disease (MOGAD) is an immune-mediated inflammatory demyelinating disorder of the central nervous system that is now recognized as a distinct entity from multiple sclerosis (MS) and aquaporin-4 immunoglobulin G-positive neuromyelitis optica spectrum disorder (AQP4-IgG-positive NMOSD). The 2023 International MOGAD Panel proposed diagnostic criteria that define MOGAD by the association of a compatible core demyelinating clinical event, MOG-IgG positivity, and exclusion of more likely alternative diagnoses, with validated cell-based assays playing a central role in serological confirmation. These criteria also emphasize that serological results should be interpreted in the context of pretest probability, clinical phenotype, MRI features, antibody titer, and assay methodology, particularly when fixed cell-based assays are used or when titers are low or not reported [1]. Pediatric MOGAD has a broad and age-dependent clinical spectrum. Acute disseminated encephalomyelitis (ADEM) is among the most frequent initial phenotypes in younger children, followed by optic neuritis and/or transverse myelitis, whereas older children and adolescents may present with overlapping brain, optic nerve, brainstem, or spinal cord involvement [2,3]. Neuroimaging may show ill-defined, asymmetric, ADEM-like T2-weighted/fluid-attenuated inversion recovery (T2/FLAIR) brain lesions, as well as spinal cord involvement, including longitudinally extensive myelitis, which can support the diagnosis when interpreted together with clinical and serological findings [1,3]. Although many pediatric cases follow a monophasic course, relapses are well recognized and remain difficult to predict, with reported relapse rates varying across cohorts [2,4]. This variability emphasizes the need for prolonged clinical surveillance, as maintenance therapy is generally considered after a first relapse in pediatric MOGAD to reduce the risk of further attacks and permanent sequelae [5].

The present case illustrates the diagnostic and follow-up challenges posed by a MOG-IgG-positive demyelinating event occurring after a prolonged symptom-free interval following an earlier ADEM-like episode in childhood. Such a presentation should prompt clinicians to consider the possibility of later recurrent demyelination, particularly when MOG-IgG testing was unavailable during the initial event. This case therefore supports careful reassessment of previous ADEM diagnoses when recurrent multifocal central nervous system demyelination occurs and emphasizes the need for long-term surveillance even after apparently complete recovery.

## Case presentation

In September 2025, a 14-year-old male adolescent, born to first-degree consanguineous parents and the second of three siblings, was admitted for acute motor weakness occurring in a febrile context. He had normal psychomotor development and normal school performance, with no reported academic difficulties. He had no known family history of neurological or autoimmune disease and was not receiving any long-term medication.

At the age of nine years, the patient had been hospitalized for a first neurological episode characterized by dorsolumbar back pain, lower limb pain, gait and balance disturbances, and urinary incontinence (Table 1). Neurological examination at that time revealed an ataxic gait, brisk deep tendon reflexes in the lower limbs, a positive Babinski sign, and neck stiffness. Neurological examination also revealed mild drowsiness. The patient was febrile, with a recorded temperature of 39°C. Lumbar puncture performed during this first episode (Table 2) showed cerebrospinal fluid pleocytosis of 53 cells/mm^3^, composed of 60% neutrophils and 40% lymphocytes, with elevated protein levels at 0.56 g/L (reference range: 0.15-0.40 g/L) and preserved cerebrospinal fluid glucose at 0.76 g/L (reference range: 0.40-0.70 g/L). Herpes simplex virus type 1 polymerase chain reaction was negative. Brain and spinal magnetic resonance imaging showed signal abnormalities involving the subcortical white matter and medulla oblongata, associated with longitudinally extensive myelitis (Figures 1A, 1B). A diagnosis of ADEM was made at that time on the basis of the overall clinico-radiological presentation, including a first polyfocal central nervous system event, mild drowsiness, multifocal brain and medullary lesions, longitudinally extensive myelitis, and cerebrospinal fluid pleocytosis. MOG-IgG and AQP4-IgG testing were not performed at that time because these tests were not available in our setting. The patient received intravenous methylprednisolone pulses at a dose of 30 mg/kg/day for five days, followed by oral corticosteroid therapy at 1 mg/kg/day for four weeks, with gradual tapering over the following six weeks. The clinical course was favorable, with complete recovery. He remained asymptomatic for five years.

**Table 1:** Chronological summary of the patient's clinical course AQP4-IgG: aquaporin-4 immunoglobulin G; MOG-IgG: myelin oligodendrocyte glycoprotein immunoglobulin G; MRI: magnetic resonance imaging

Time point	Clinical presentation	Main investigations	Treatment	Outcome
Age nine years	First demyelinating episode with fever, dorsolumbar back pain, gait and balance disturbance, urinary incontinence, mild drowsiness, pyramidal signs, and neck stiffness.	Cerebrospinal fluid pleocytosis and elevated protein level; brain and spinal MRI showing multifocal brain lesions and longitudinally extensive myelitis; MOG-IgG and AQP4-IgG testing not available.	Intravenous methylprednisolone followed by oral corticosteroid tapering.	Complete clinical recovery.
Following five years	No neurological symptoms.	No relapse reported.	No maintenance immunotherapy.	Prolonged symptom-free interval.
Age 14 years	Acute febrile episode with severe quadriparesis predominating in the lower limbs, pyramidal signs, and sphincter dysfunction.	Brain MRI showing multifocal poorly demarcated subcortical lesions; spinal MRI showing longitudinally extensive cervical myelitis; cerebrospinal fluid marked pleocytosis; serum MOG-IgG positive; AQP4-IgG negative; no oligoclonal bands.	Intravenous methylprednisolone followed by intravenous immunoglobulin because of insufficient initial improvement.	Progressive motor and sphincter recovery.
One-month follow-up	No objective motor deficit; independent walking; resolution of sphincter dysfunction.	Clinical examination normalized.	Rehabilitation continued; mycophenolate mofetil maintenance therapy initiated.	Complete clinical recovery.
Six-month follow-up	No clinical relapse.	Follow-up MRI showing marked regression of brain and spinal cord lesions; persistent serum MOG-IgG positivity.	Continued mycophenolate mofetil with clinical and biological monitoring.	Favorable short-term clinical and radiological course.

**Table 2:** Comparative cerebrospinal fluid, infectious, and immunological findings during the first and current attacks AQP4-IgG: aquaporin-4 immunoglobulin G; CSF: cerebrospinal fluid; HSV: herpes simplex virus; IgG: immunoglobulin G; MOG-IgG: myelin oligodendrocyte glycoprotein immunoglobulin G; NMOSD: neuromyelitis optica spectrum disorder; PCR: polymerase chain reaction

Investigation	First attack, at age nine years	Current attack, at age 14 years	Interpretation
CSF appearance	Clear	Clear	Normal appearance
CSF leukocyte count	53 cells/mm^3^	275 cells/mm^3^	Pleocytosis during both attacks, more marked during the current episode
CSF differential count	60% neutrophils; 40% lymphocytes	40% neutrophils; 60% lymphocytes	Mixed pleocytosis
CSF red blood cells	15 cells/mm^3^	30 cells/mm^3^	Low red blood cell count during the current episode
CSF protein level	0.56 g/L	1.16 g/L	Elevated during both attacks
CSF glucose level	0.76 g/L	0.70 g/L	Preserved CSF glucose
HSV PCR in CSF	Negative for HSV-1	Negative for HSV-1	HSV infection not detected
CSF direct examination/microscopy	Negative	Negative	No organisms detected during either attack
CSF bacterial culture	Negative	Negative	No bacterial growth during either attack
Blood cultures	Negative	Negative	No bacterial growth during either attack
Serum MOG-IgG	N/A	Positive, titer 1:100 by fixed cell-based assay using MOG-transfected cells (Euroimmun)	Serological confirmation of MOG-IgG positivity during the current episode
Serum AQP4-IgG	N/A	Negative	AQP4-IgG-associated NMOSD unlikely
CSF and serum isoelectric focusing	N/A	Type 1 pattern	No CSF-restricted oligoclonal bands
IgG index	N/A	0.489	No evidence of intrathecal IgG synthesis

**Figure 1:**
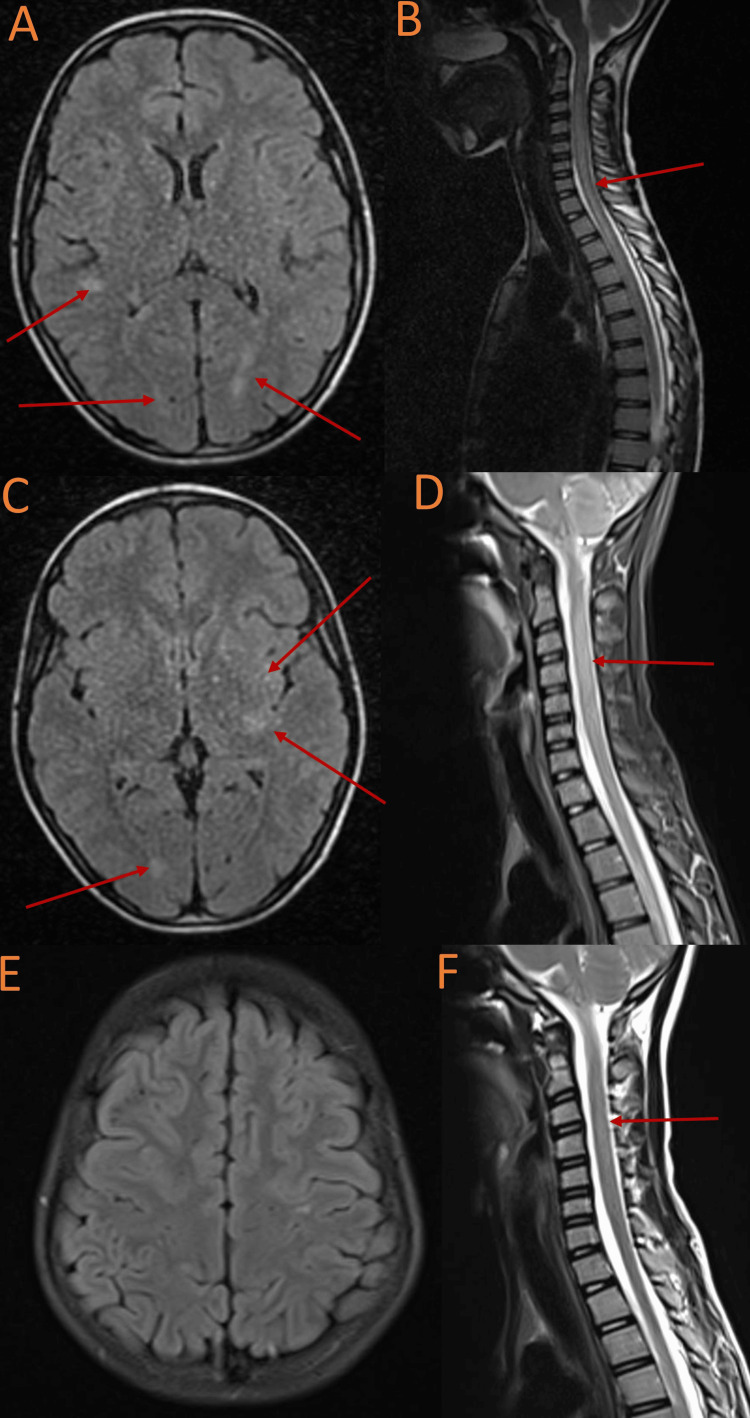
Brain and spinal MRI findings during the initial demyelinating episode, the current relapse, and follow-up (A) Axial brain FLAIR MRI during the first episode showing multifocal ill-defined supratentorial subcortical white matter hyperintensities (red arrow), supporting an inflammatory ADEM-like demyelinating process. (B) Sagittal cervicothoracic STIR MRI showing a longitudinally extensive central intramedullary hyperintensity (red arrow), without extrinsic compression, consistent with inflammatory myelitis. (C) Axial brain FLAIR MRI during the current relapse showing recurrent poorly demarcated supratentorial white matter lesions (red arrow); in the clinical and serological context, this pattern favored MOGAD over typical pediatric-onset multiple sclerosis. (D) Sagittal cervical T2-weighted MRI showing longitudinally extensive central cervical cord hyperintensity with mild swelling (red arrow), supporting recurrent inflammatory myelitis and arguing against a short peripheral multiple sclerosis-type lesion. (E) Six-month follow-up axial brain FLAIR MRI showing marked regression of the supratentorial lesions, without new lesions, supporting the inflammatory and reversible nature of the abnormalities. (F) Six-month follow-up sagittal cervical T2-weighted MRI showing marked improvement of the previously extensive cord lesion (red arrow), consistent with favorable radiological evolution in pediatric MOGAD. ADEM: acute disseminated encephalomyelitis; FLAIR: fluid-attenuated inversion recovery; MOGAD: myelin oligodendrocyte glycoprotein antibody-associated disease; MOG-IgG: myelin oligodendrocyte glycoprotein immunoglobulin G; MRI: magnetic resonance imaging; STIR: short tau inversion recovery

The current episode began three days before admission with an influenza-like febrile illness and a recorded temperature of 39°C. The following day, the patient developed gait disturbance associated with sphincter dysfunction in the form of urinary incontinence. Twenty-four hours later, his condition rapidly worsened, with complete inability to move both lower limbs and persistent fever. He did not report headache, vomiting, photophobia, back pain, recent weight loss, anorexia, or known contact with tuberculosis. On admission, the patient was mildly confused, with a Glasgow Coma Scale score of 14/15. He was hemodynamically and respiratorily stable, with a blood pressure of 110/75 mmHg, heart rate of 110 beats/min, respiratory rate of 18 breaths/min, peripheral oxygen saturation of 97%, capillary blood glucose level of 0.95 g/L, and body temperature of 40°C. Neurological examination revealed that standing was impossible, and sitting was possible only with assistance. Muscle strength, assessed using the Medical Research Council (MRC) scale [6], was 0/5 in both lower limbs, 0/5 in the left upper limb, and 2/5 in the right upper limb. Muscle tone was increased, with a pyramidal pattern predominating in the lower limbs. Deep tendon reflexes were brisk in all four limbs, with bilateral Babinski signs and bilateral exhaustible Achilles clonus. Sensory examination revealed no clearly identifiable sensory level, with preservation of both superficial and deep sensations. Cranial nerve examination was unremarkable. There was no nystagmus, diplopia, facial asymmetry, dysarthria, or swallowing disorder. Cerebellar examination was limited by the severe motor deficit, as standing and walking were impossible. Initial ophthalmological examination showed no evidence suggestive of optic neuritis. Visual acuity was preserved at 10/10 in both eyes, color vision was normal, and direct and consensual pupillary light reflexes were present and symmetrical. There was no relative afferent pupillary defect. Fundoscopic examination was normal.

Given this clinical presentation, urgent brain and spinal magnetic resonance imaging was performed to exclude a compressive emergency, including tumoral or infectious causes, and to investigate an inflammatory demyelinating disorder of the central nervous system, particularly MS, NMSOD, or MOGAD. Brain MRI showed multiple patchy and nodular signal abnormalities, disseminated, ill-defined, asymmetric, and variable in size, involving the subcortical white matter. These lesions were hyperintense on T2-weighted and T2-FLAIR sequences, without diffusion restriction or gadolinium enhancement. They were located in the right centrum semiovale, left capsular region, bilateral frontoparietal regions, and bilateral occipital regions (Figure 1C). Spinal MRI showed a longitudinally extensive central spinal cord signal abnormality extending from the cervicomedullary junction to C6, associated with spinal cord swelling. The lesion was isointense on T1-weighted imaging, hyperintense on T2-weighted imaging, and enhanced after gadolinium administration. The conus medullaris was normally positioned at the T12 level. The paravertebral soft tissues were preserved, with no radiological evidence of spinal cord compression or spondylodiscitis (Figure 1D).

Initial laboratory investigations showed no evidence of systemic inflammation, with a normal complete blood count and a C-reactive protein level of 2 mg/L (normal: <5 mg/L). Cerebrospinal fluid analysis showed a clear appearance, pleocytosis of 275 leukocytes/mm^3^ (normal: <5 cells/mm^3^), composed of 40% neutrophils and 60% lymphocytes, with 30 red blood cells/mm^3^, elevated protein levels at 1.16 g/L (reference range: 0.15-0.40 g/L), normal glucose level at 0.70 g/L (reference range: 0.40-0.70 g/L), and negative direct cerebrospinal fluid examination. Herpes simplex virus polymerase chain reaction was negative. Additional infectious investigations, including blood cultures and cerebrospinal fluid bacterial culture, were negative (Table 2). Given the inflammatory appearance on MRI, the longitudinally extensive myelitis, and the previous ADEM-like demyelinating episode, an immunological workup was performed. Serum MOG-IgG was positive at a titer of 1:100 using a fixed cell-based assay with MOG-transfected cells (Euroimmun, Lübeck, Germany). Anti-aquaporin-4 IgG antibodies were negative. Cerebrospinal fluid and serum isoelectric focusing showed a type 1 pattern, with no intrathecal oligoclonal bands. The IgG index was normal at 0.489, arguing against intrathecal IgG synthesis.

The combination of longitudinally extensive myelitis, absence of oligoclonal bands, positivity of anti-myelin oligodendrocyte glycoprotein antibodies, and negativity of anti-aquaporin-4 antibodies argued against MS and made anti-aquaporin-4 antibody-associated NMSOD unlikely. The final diagnosis for the current episode was MOG-IgG-positive MOGAD presenting as ADEM-like encephalomyelitis associated with longitudinally extensive cervical myelitis. Because MOG-IgG testing had not been performed during the first episode, the current event was considered a confirmed MOG-IgG-positive demyelinating event occurring after a previous ADEM-like episode, rather than definitive proof that the first episode had been MOGAD.

The patient received intravenous methylprednisolone pulses at a dose of 30 mg/kg/day for five days. Because no significant clinical improvement was observed after this course of intravenous corticosteroid therapy, intravenous immunoglobulin therapy was initiated at a total dose of 2 g/kg, administered over five days, corresponding to 0.4 g/kg/day. Progressive motor improvement was observed from the third day of intravenous immunoglobulin therapy, with partial recovery first in the upper limbs and subsequently in the lower limbs. At the end of the intravenous immunoglobulin course, muscle strength was graded as 4/5 in the right upper limb, 3/5 in the left upper limb, 2/5 in the proximal muscles of both lower limbs, and 3/5 in the distal muscles of both lower limbs. Sphincter dysfunction gradually improved, with resumption of spontaneous urination after a few days. Temporary urinary catheterization was required during the acute phase because of severe bladder dysfunction and the risk of urinary retention. The patient underwent intensive motor rehabilitation. No significant adverse effects related to corticosteroid or intravenous immunoglobulin therapy were observed. Maintenance therapy with mycophenolate mofetil was initiated at a total dose of 1200 mg/m^2^/day, divided into two daily doses, after an unremarkable pretreatment assessment. Clinical and biological monitoring was planned during treatment.

One month after the acute episode, the clinical course was favorable. The patient had no objective motor deficit, with muscle strength graded as 5/5 in all four limbs according to the MRC scale. He was able to walk independently, without assistance or limping, and standing was stable. Sphincter dysfunction had completely resolved, with normal spontaneous urination and no urinary incontinence. Neurological examination showed no sensory deficit, cranial nerves were intact, and ophthalmological examination remained normal, with visual acuity preserved at 10/10 in both eyes. The patient continued outpatient rehabilitation and gradually resumed his daily activities, including school attendance.

A follow-up brain and spinal MRI performed six months later showed marked regression of the subcortical white matter lesions and cervical central spinal cord abnormalities (Figures 1E, 1F). Serum MOG-IgG antibodies remained positive. A structured long-term surveillance plan was established, including regular neurological and ophthalmological assessments, monitoring of mycophenolate mofetil tolerance, repeat brain and spinal MRI, and serial serum MOG-IgG testing when available.

## Discussion

MOGAD is an inflammatory demyelinating disorder of the central nervous system that is now considered distinct from MS and aquaporin-4 antibody-positive NMSOD [1]. Although MOG antibodies had been described earlier, their clinical relevance became clearer with the development of cell-based assays, which improved the detection of conformational MOG-IgG and helped define MOGAD as a separate disease entity [7]. MOGAD is rare, but it represents an important cause of acquired demyelinating syndromes in children. Available epidemiological data suggest that MOGAD is uncommon, with reported prevalence estimates of approximately 1.3-2.5 per 100,000 and annual incidence estimates of approximately 3.4-4.8 per million [8]. The clinical phenotype is age-dependent. In younger and prepubertal children, ADEM is one of the most frequent presentations, whereas optic neuritis and transverse myelitis become more common in older children and adolescents [2,9]. Our patient’s first episode at nine years was diagnosed as ADEM, while the second episode at 14 years was MOG-IgG-positive and dominated by severe longitudinally extensive cervical myelitis. This temporal evolution is compatible with the age-dependent clinical spectrum of pediatric MOGAD; however, the first episode cannot be definitively attributed to MOGAD because MOG-IgG testing was not available at that time. Therefore, the first attack is described as an ADEM-like or ADEM-compatible demyelinating episode rather than as confirmed MOGAD. Although many children with MOGAD have a monophasic course, relapses are well recognized. Pediatric relapsing phenotypes include multiphasic disseminated encephalomyelitis, ADEM followed by optic neuritis, relapsing optic neuritis, and opticospinal or myelitic presentations [9]. Most relapses occur within the first few years after onset, but delayed relapses have also been reported. In one multicenter cohort of relapsing MOGAD, a first relapse occurring five years or more after the initial event was documented in a subset of patients [10]. In our patient, the five-year symptom-free interval after the initial ADEM-like episode is unusual and highlights the need for prolonged clinical vigilance after pediatric demyelinating events, particularly when recurrent multifocal central nervous system demyelination occurs.

The diagnostic interpretation of the current episode was based on the combination of a compatible demyelinating clinical syndrome, characteristic MRI features, inflammatory cerebrospinal fluid findings, serum MOG-IgG positivity, and exclusion of alternative diagnoses, in accordance with the 2023 International MOGAD Panel criteria [1]. The multifocal, poorly demarcated brain lesions and longitudinally extensive myelitis were consistent with recognized pediatric MOGAD imaging patterns [3]. The cerebrospinal fluid profile supported an inflammatory demyelinating process, particularly because pleocytosis was marked and cerebrospinal fluid-restricted oligoclonal bands were absent. Although cerebrospinal fluid protein was elevated, this parameter should be interpreted cautiously, as protein levels do not consistently correlate with clinical severity in MOGAD and should not be used alone to estimate prognosis [11]. Serum MOG-IgG was detected at a titer of 1:100 using a fixed cell-based assay with MOG-transfected cells (Euroimmun). Current diagnostic frameworks emphasize serum testing with validated cell-based assays, and live cell-based assays using full-length human MOG are generally considered the preferred method when available [1]. In our patient, the fixed cell-based assay result was interpreted in the context of a high pretest probability phenotype, including longitudinally extensive myelitis, multifocal poorly demarcated brain lesions, absence of oligoclonal bands, negative AQP4-IgG, and exclusion of infectious and compressive causes. A confirmatory live cell-based assay would have further strengthened the serological evidence, but was not available in our setting. The absence of MOG-IgG testing during the first attack remains a limitation and prevents definitive retrospective classification of that episode. However, the current event fulfilled diagnostic criteria for MOG-IgG-positive MOGAD when interpreted in the full clinical, radiological, and biological context. Several features favored MOGAD over pediatric-onset MS and AQP4-IgG-positive NMOSD. The brain lesions were multifocal, ill-defined, asymmetric, and predominantly subcortical, without typical MS features such as multiple small well-demarcated ovoid periventricular lesions, Dawson-finger-like morphology, prominent corpus callosum involvement, chronic T1 “black holes,” or dissemination in time with persistent new silent lesions. The spinal cord lesion was longitudinally extensive and centrally located with cord swelling, which is more compatible with MOGAD or NMOSD than with typical MS, in which spinal lesions are often shorter and more peripheral. Compared with AQP4-IgG-positive NMOSD, our patient had negative AQP4-IgG, no optic chiasm involvement, no area postrema syndrome, and no characteristic periependymal/diencephalic pattern. The marked regression of both brain and spinal cord lesions on follow-up MRI also supports MOGAD, because pediatric MOGAD lesions are more likely to resolve or substantially regress over time than lesions in pediatric-onset MS or AQP4-IgG-positive NMOSD [3,12].

High-dose intravenous corticosteroids are generally considered the first-line treatment for acute MOGAD attacks. In severe attacks or in cases with insufficient response to intravenous methylprednisolone, intravenous immunoglobulin or plasma exchange may be considered as rescue therapies [5]. This approach was applied to our patient, who had severe cervical myelitis with major motor impairment and sphincter dysfunction. Because no significant improvement was observed after five days of intravenous methylprednisolone, intravenous immunoglobulin therapy was administered at a total dose of 2 g/kg over five days, followed by progressive motor and sphincter recovery. The decision to start maintenance therapy was guided by the severity of the second attack and the highly probable relapsing course. Pediatric MOGAD does not always require long-term immunosuppression after a first event, as many cases are monophasic. However, the E.U. pediatric MOG consortium recommends considering maintenance treatment after a first relapse to reduce the risk of further relapses and possible permanent neurological sequelae [5]. Mycophenolate mofetil is among the first-line maintenance options proposed, together with azathioprine and monthly intravenous immunoglobulins, whereas tocilizumab has been reported for refractory cases. Rituximab is also used as a steroid-sparing maintenance therapy, although relapse prevention may be variable in MOGAD compared with AQP4-IgG-positive NMOSD [5]. In this patient, the decision to initiate maintenance therapy was individualized and guided by the severity of the confirmed MOG-IgG-positive episode, the major motor and sphincter impairment during the acute phase, the previous ADEM-like demyelinating episode, persistent MOG-IgG positivity, and the concern for further disabling attacks. Mycophenolate mofetil was selected because it was available in our setting, feasible as a long-term oral steroid-sparing therapy, familiar to our institution, and compatible with regular clinical and biological monitoring. Despite the severity of the acute presentation, the outcome in our patient was favorable, with complete motor and sphincter recovery one month after the relapse and marked regression of both brain and cervical spinal cord lesions on follow-up MRI at six months. This evolution illustrates a recognized feature of MOGAD: acute attacks may be clinically and radiologically severe, particularly when associated with longitudinally extensive myelitis, but functional recovery is often good when treatment is initiated promptly [2,5]. Radiological improvement also supports the diagnosis, as pediatric MOGAD lesions are more likely to regress or resolve on follow-up MRI than lesions seen in AQP4-IgG-positive NMOSD or MS. In a pediatric comparative MRI study, T2 lesion resolution was more frequent in MOGAD than in AQP4-positive NMOSD and MS, both in the brain and spinal cord [12]. This pattern is consistent with our patient, whose six-month MRI showed marked regression of the cerebral and cervical spinal cord lesions. However, the available follow-up after initiation of mycophenolate mofetil remains short. The absence of relapse during six months, together with radiological improvement, is encouraging but insufficient to establish long-term disease control or to determine the effectiveness of mycophenolate mofetil in this individual patient. Longer clinical and imaging surveillance is required.

Long-term surveillance is essential in pediatric MOGAD because relapse risk is difficult to predict and delayed relapses may occur even after several years of clinical stability. Recent French guidelines recommend long-term follow-up in a reference or expert center at least once a year, with interim follow-up every six months by the treating pediatric neurologist or neurologist. Follow-up should assess new neurological, visual, cognitive, motor, or bladder/sphincter symptoms, treatment adherence and tolerance, and the need for rehabilitation or multidisciplinary support [13]. MRI surveillance should be individualized, but recent MRI management proposals for MOGAD suggest brain, optic nerve, and spinal cord MRI at diagnosis, a control MRI at approximately six months focusing on the involved area, and, in clinically inactive patients, gadolinium-free brain and spinal cord MRI every 36 months [14]. When available, serial serum MOG-IgG testing may also be useful; the E.U. pediatric MOG consortium recommends retesting MOG-IgG every six months until two years from onset or until seronegative conversion [15]. In our patient, this supports continued neurological and ophthalmological follow-up, treatment-tolerance monitoring under mycophenolate mofetil, repeat brain and spinal MRI, and reassessment of serum MOG-IgG status during follow-up.

## Conclusions

This case shows that a MOG-IgG-positive demyelinating event may occur after a five-year relapse-free interval following an earlier ADEM-like episode in childhood. Because MOG-IgG testing was unavailable during the first attack, that episode should be interpreted cautiously as ADEM-like rather than confirmed MOGAD. In adolescents with recurrent multifocal demyelination and longitudinally extensive myelitis, MOGAD should be reconsidered even after years of apparent clinical stability. Favorable short-term clinical and radiological recovery does not establish durable disease control, and prolonged neurological, ophthalmological, imaging, and treatment-tolerance surveillance remains necessary.
